# 冠状动脉支架植入后肺癌患者行肺切除术的围手术期结局

**DOI:** 10.3779/j.issn.1009-3419.2020.01.06

**Published:** 2020-01-20

**Authors:** 伟明 黄, 康 齐, 志茂 陈, 简 李

**Affiliations:** 100034 北京，北京大学第一医院胸外科 Department of Thoracic Surgery, Peking University First Hospital, Beijing 100034, China

**Keywords:** 肺肿瘤, 冠状动脉疾病, 手术, 并发症, 抗血小板治疗, Lung neoplasms, Coronary artery disease, Surgery, Complications, Antiplatelet therapy

## Abstract

**背景与目的:**

肺癌患者合并有冠心病是很常见的一种情况，部分患者既往植入冠脉支架并接受抗血小板治疗。对于带有冠脉支架需要行肺切除手术的肺癌患者，围手术期是否停用抗血小板药物仍然存在争议。本研究通过回顾我院的数据来明确这部分特殊人群的围手术期结局。

**方法:**

回顾性分析了2013年1月-2019年9月冠脉支架术后在北京大学第一医院胸外科行肺切除手术的肺癌患者的临床数据。所有患者术前暂停口服抗血小板药物至少5 d。主要研究终点是院内的心血管并发症和死亡。

**结果:**

本研究共纳入111例患者。支架放置和肺癌手术间隔的时间在1个月-3个月、3个月-12个月、超过12个月的患者分别为6.3%、13.5%和80.2%。亚肺叶切除、肺叶切除、联合肺叶切除、全肺切除以及肺叶袖式切除的患者分别为10.8%、71.2%、9.0%、2.7%和6.3%。总的心血管并发症发生率为11.6%，包括不稳定心绞痛（*n*=1, 0.9%）、低血压（*n*=1, 0.9%）、充血性心力衰竭（*n*=2, 1.8%）和新发心房纤颤（*n*=10, 9.0%）。本组病例无围手术期死亡，无主要不良心血管事件（major adverse cardiac events, MACE）。

**结论:**

术前暂停口服抗血小板药物是安全的，围手术期未发生MACE和死亡。

## Abstract (en)

**Background and objective:**

It is common for patients with lung cancer to have concomitant coronary artery disease, some of them have underwent coronary stenting and accepted antiplatelet therapy. Whether antiplatelet agents should be continued in the perioperative period remains controversial, especially in patients requiring lung cancer resection with coronary artery stents. We reviewed the data of our institute to clarify the perioperative outcomes of this specific population.

**Methods:**

We retrospectively analyzed the data on patients who underwent pulmonary resection for lung cancer following coronary stent placement between January 2013 and September 2019 in the Department of Thoracic Surgery in Peking University First Hospital. All of them discontinued oral antiplatelet agents before operation at least 5 days. The primary outcomes were the cardiovascular morbidity and mortality in hospital.

**Results:**

111 patients were identified and included in the analysis. The time intervals between stenting and lung surgery were 1-3 months, 3-12 months, and more than 12 months in 6.3%, 13.5% and 80.2% of the patients, respectively. Sublobectomy, lobectomy, biolobectomy, pneumonectomy and sleeve lobectomy were performed in 10.8%, 71.2%, 9.0%, 2.7% and 6.3% of the patients. The overall incidences of cardiovascular complications were 11.6%, including unstable angina (*n*=1, 0.9%), hypotention (*n*=1, 0.9%), congestive heart failure (*n*=2, 1.8%) and new-onset atrial fibrillation (*n*=10, 9.0%). There was no perioperative death. No major adverse cardiac events (MACE) occurred.

**Conclusion:**

It was safety to discontinued oral antiplatelet agents before operation, with no MACE and death in perioperative period.

肺癌患者合并有冠心病是很常见的一种情况^[1]^。部分接受手术的患者既往植入冠脉支架，并长期服用抗血小板药物，围手术期需要权衡停药后的心血管不良事件与持续用药的出血风险。研究提示停用抗血小板药物会增加支架内血栓的风险^[2]^，但是围手术期持续抗血小板治疗，会增加出血的风险^[3]^。冠脉支架植入后行非心脏手术的患者，围手术期的抗血小板治疗策略到目前仍无定论^[4]^。最近的指南建议是根据患者具体情况由心内科、外科和麻醉科等多科会诊制定个体策略^[5]^，没有针对胸外科或肺肿瘤的专门建议。

回顾文献胸部及肺肿瘤外科手术合并冠脉支架的研究很少，多为30例左右的小样本研究，而且争议同样存在^[6, 7]^。对于这部分特殊患者人群，本中心的临床习惯是在围手术期暂停口服抗血小板药物。本研究为单中心回顾性研究，通过分析2013年1月-2019年9月在我中心带有冠状动脉支架的肺癌手术患者的数据，明确这部分人群暂停抗血小板药物的围手术期结局，为今后的临床工作和研究提供依据。

## 1 资料与方法

### 1.1 临床资料

回顾性分析了北京大学第一医院胸外科从2013年1月-2019年9月带有冠状动脉支架的并接受手术的全部肺癌患者数据。本中心所有患者术前均停用抗血小板药物至少5 d，研究的主要终点是院内的心血管并发症和死亡。主要不良心血管事件（major adverse cardiac events, MACE）的定义参照既往研究^[8]^提出的包括心源性死亡、心脏停搏、ST段抬高和ST段不抬高的心梗、支架内血栓以及通过冠脉搭桥手术、支架及血管成形等冠脉再次血管化治疗。其他的数据包括手术情况、抗血小板药物的种类和暂停时间以及是否用低分子肝素药物进行桥接等也一并统计。

### 1.2 统计学方法

采用统计软件SPSS 23.0进行数据处理分析。本研究描述性统计中，定量变量用Mean±SD或中位数表示，定性变量用频数和百分比表示。

## 2 结果

### 2.1 一般资料

共收集病例111例，其中男性89例，女性22例（表 1）。平均年龄67.4岁（47岁-82岁），超过半数的患者合并有其他内科疾病，最常见的是高血压和糖尿病，有36例患者既往有心肌梗塞的病史。支架植入距肺切除手术的时间，没有 < 1个月的病例，3个月内的有7例，89例（80.2%）在12个月以上。近半数患者留置多枚支架，平均2.0枚。

**1:** 患者的一般资料（*n*=111） Patient demographics (*n*=111)

Variable		Data
Age [yr, Mean±SD (range)]		67.4±7.7 (47-82)
Gender	Male Female	89 (80.2%) 22 (19.8%)
Comorbidities	Diabetes mellitus Hypertension Peripheral vascular disease Atrial Fibrillation History of cerebrovascular diseases	42 (37.8%) 60 (54.1%) 7 (6.3%) 7 (6.3%) 9 (8.1%)
History of myocardial infarction		36 (32.4%)
Ejection fraction < 50 %		5 (4.5%)
Time from last stent implantation to operation	1-3 months 3-12 months 12-36 months > 36 months	7（6.3%) 15 (13.5%) 30 (27.0%) 59 (53.2%)
Number of stents	1 2 3 > 3 Unknown	51 (45.9%) 18 (16.2%) 15 (13.2%) 12 (10.8%) 15 (13.5%)

### 2.2 手术结果

采用手术入路中全胸腔镜下切除76例，保留肌肉神经的小切口34例，正中开胸1例为左肺上沟瘤切除术。接受亚肺叶（包括楔形和肺段）切除的12例，肺叶切除79例，联合肺叶切除10例，全肺切除3例以及支气管袖式切除7例，大部分患者（*n*=96, 86.5%）完成纵隔淋巴结清扫（表 2）。明显粘连（大片状、非仅条索样）29例，其中同侧2次开胸手术的2例。术中出血中位数100 mL，手术中位时间227 min，术中需要输血1例。

**2:** 手术相关因素和结局 Operative procedure-related factors and outcomes

Variable		Data
Approach	VATS Thoracotomy	76 (68.5%) 35 (31.5%)
Procedure	Sublobectomy Lobectomy Bilobectomy Pneumonectomy Sleeve Lobectomy	12 (10.8%) 79 (71.2%) 10 (9.0%) 3 (2.7%) 7 (6.3%)
Mediastinal LND		96 (86.5%)
Adhesion		29 (26.1%)
Re do thoracotomy		2 (1.8%)
Histology	AIS/MIA AC SCC Others	8 (7.2%) 59 (53.2%) 32 (28.8%) 12 (10.8%)
Intraoperative bleeding (mL)		100 (5-1, 100)
Length of operation (min)		227 (51-611)
Blood transfusion		1 (0.9%)
VATS: video-assisted thoracic surgery; LND: lymph node dissection; AIS: adenocarcinoma *in situ*; MIA: minimally invasive adenocarcinoma; AC: adenocarcinoma; SCC: squamous cell carcinoma.		

### 2.3 抗血小板治疗状况

70例患者采用口服单药抗血小板治疗，37例采用双联抗血小板治疗（表 3）。术前均停药5 d以上，停药时间在2周内共87例，其中1周内的61例。超过2周的有20例，是因为术前门诊气管镜或穿刺活检停药后叠加手术计划，患者未再重新服用药物。另外4例长时间停药超过半年，其中2例是因为间断咯血停药，2例自行停药。72例患者术前采用低分子肝素进行抗凝桥接替代抗血小板药物。术后72 h后根据引流情况恢复抗血小板治疗。

**3:** 围手术期抗血小板治疗 Perioperative management of antiplatelet therapy

Variable		Data
Time from discontinuation of antiplatelet agents to operation	5-7 days 8-14 days > 14 days	61 (55.0%) 26 (23.4%) 20 (18.0%)
Oral antiplatelet agents	Aspirin Clopidogrel or ticagrelor Dual antiplatelet None	58 (52.3%） 12 (10.8%） 37 (33.3%） 4 (3.6%）
Use LMWH for bridging	Yes No	72 (64.9%) 39 (35.1%)
LMWH: low molecular weight heparin.		

### 2.4 心血管并发症

心血管并发症总发生率为11.6%。其中1例患者术后第2天出现不稳定心绞痛，持续约15 min后缓解，不伴有心肌酶的升高，没有构成急性心梗的诊断。1例患者因术前就合并病窦综合征，术后出现一过性心率减慢及血压降低，未出现心脏停搏，经药物治疗后短时间好转。2例患者出现充血性心衰症状，经利尿扩血管治疗后好转。有10例术后新发房颤。无术后肺栓塞病例。全部病例中无院内死亡和MACE，即心源性死亡、心脏停搏、ST段抬高和ST段不抬高的心梗、支架内血栓等（表 4）。

**4:** 围手术期心血管并发症 Perioperative cardiovascular complications

Variable	Data
Death in hospital	0 (0.0%)
Major adverse cardiac events	0 (0.0%)
Unstable angina	1 (0.9%)
New-onset atrial fibrillation	10 (9.0%)
Hypotention	1 (0.9%)
Congestive heart failure Pulmonary embolism	2 (1.8%) 0 (0.0%)

## 3 讨论

本研究为单中心回顾性观察研究，通过分析2013年1月-2019年9月所有111例支架术后因肺癌行手术的患者人群的数据，明确暂停抗血小板药物后围手术期间的心血管并发症的发生率。本研究结果显示该人群的性别分布上，男性远多于女性（80.2% *vs* 19.8%），和Fernandez^[8]^等采集美国国立癌症研究所监测、流行病学和结果数据库（Surveillance, Epidemiology and End Results, SEER）的结果一致。本组病例中无死亡和MACE。只有1例和冠脉缺血相关的并发症，即不稳定心绞痛，持续15 min缓解。1例出现心率减慢、血压降低，和患者本身的病窦综合征相关，其他包括心衰和新发的房颤经证实与冠状动脉缺血无关。

冠心病相关的研究表明恶性肿瘤是支架内血栓的独立危险因素^[9]^，停用抗血小板药物也会增加支架内血栓的风险^[2]^，支架植入距离手术时间越短，MACE尤其是支架内血栓的发生率越高^[10]^。Brichon等^[11]^回顾分析了32例裸支架术后3个月内行肺切除手术的病例，阿司匹林单药维持围术期，仍有3例（9%）出现支架内血栓，远高于同期未进行手术的0.5%。本研究的患者大部分支架植入超过1年（80.2%），3个月内的只有6.3%，没有1个月内的病例。这可能也是本研究中没有MACE发生的一个重要原因。本研究中的1例术后不稳定心绞痛的患者，支架术后距手术时间为9个月。

是否应当在围手术期持续口服抗血小板药物，研究结论并不一致。多数研究证实术前持续口服抗血小板药物，会显著增加外科手术包括肺切除手术的出血风险和围手术期输血概率^[3, 4, 11-13]^，也有研究^[14]^认为不会增加出血风险。暂停抗血小板药物是否会增加围手术期MACE的发生，研究结果相差比较大，尤其体现在欧美和亚洲之间。来自美国的Cerfolio^[12]^和Atay^[15]^等的研究中亚组分析带有支架行肺叶切除的患者，停药后MACE发生率分别为36%和8.5%。而来自亚洲的研究包括韩国的Yu^[13]^、日本的Sonobe^[16]^以及中国的李昕^[17]^等停用抗血小板药物后均未出现MACE。以上研究的局限在于样本数目少，每个研究最多总数也不超过35例。Sonobe等^[16]^提出从冠状动脉疾病数据库看，相对于欧美人群，日本人群冠脉支架血栓发生率要低得多，30 d内裸支架和药物洗脱支架分别为0.9%和0.34%，而欧美人群分别为3.6%和2.4%。认为这是欧美和日本研究结果差异的原因。并建议日本患者术前暂停抗血小板药物。

带有冠脉支架又需要接受手术的肺癌患者逐年增多，但缺乏大样本研究数据。我们发现，无论研究的结论是否支持暂停抗血小板药物，从研究方法描述中还是能看到从欧美到亚洲在实际临床工作中，多数胸外科患者术前暂停口服抗血小板药物，反映了胸外科医生对围手术期出血风险的担忧和厌恶^[6, 7, 13, 15, 16]^。在我们的研究中，带有冠脉支架又需要接受手术的肺癌患者术前暂停口服抗血小板药物是安全的，围手术期没有发生MACE和死亡。本研究的手术操作中肺叶及更复杂的切除手术占了89.2%，纵隔淋巴结清扫86.5%，在肺癌切除手术中是有一定代表性的。本研究只有1例不稳定心绞痛的发生是和冠脉缺血相关的，尽管我们研究中有部分采用低分子肝素桥接抗凝，仍然没有足够的效能来比较说明低分子肝素的作用效果，同样也无法比较不同停用抗血小板药物时间对结局的影响。

本研究只是单中心的回顾性研究，虽然围手术期的数据完整，但是由于部分患者支架植入时间较久远，缺乏患者冠状动脉支架植入手术的完整资料，无法对患者支架内血栓的危险度进行分层评估。另外，本研究大部分为支架植入1年后手术的患者，虽然有利于减少MACE的发生，但对支架早期行肺癌手术的代表性较弱。此外随着近年新型药物洗脱支架和抗血小板药物的更新换代，我们将来可能还需要多中心注册制的真实世界研究为临床决策提供更高级别的证据。
